# Involvement of oxidative modification of proteins related to ATP synthesis in the left ventricles of hamsters with cardiomyopathy

**DOI:** 10.1038/s41598-017-08546-1

**Published:** 2017-08-23

**Authors:** Sahoko Ichihara, Yuka Suzuki, Jie Chang, Kentaro Kuzuya, Chisa Inoue, Yuki Kitamura, Shinji Oikawa

**Affiliations:** 1 Graduate School of Regional Innovation Studies, Tsu, Japan; 2 Department of Human Functional Genomics, Life Science Research Center, Mie University, Tsu, Japan; 3 Department of Environmental and Molecular Medicine, Mie University Graduate School of Medicine, Tsu, Japan; 4 Department of Environmental and Preventive Medicine, Present Address: Jichi Medical University School of Medicine, Shimotsuke, Japan; 5 Present Address: Community-University Research Cooperation Center, Mie University, Tsu, Japan; 6 Present Address: School of Public Health, Medical College of Soochow University, Suzhou, China

## Abstract

Inflammation enhanced by accumulation of reactive oxygen species plays an essential role in the progression of cardiovascular diseases. Using the 2D-oxyblot analysis and 2D-difference image gel electrophoresis (2D-DIGE), we compared the levels of ROS-induced carbonyl modification of myocardial proteins in the whole left ventricles between 6-week-old hamsters with dilated (TO-2) and hypertrophic cardiomyopathy (Bio14.6) and control hamsters (F1B). Then, 2D electrophoresis combined with MALDI-TOF/TOF tandem mass spectrometry detected 18 proteins with increased carbonyl level in cardiomyopathy hamsters compared with control hamster. Carbonyl modification of proteins related to ATP synthesis, including citric acid cycle and electron transport system, was observed in the hearts of hamsters with both types of cardiomyopathy. Further analysis indicated that left ventricular carbonyl production correlated negatively with succinyl-CoA:3-ketoacid-coenzyme A transferase 1 activity (*r* ^2^ = 0.60, *P* = 0.0007) and ATP concentration (*r* ^2^ = 0.29, *P* = 0.037), suggesting that protein carbonylation has negative effects on the levels of these biomolecules. Furthermore, carbonyl production significantly correlated with plasma Troponin T level (*r* ^2^ = 0.33, *P* = 0.026). Reduction of energy metabolism by oxidative damage may contribute to the development of left ventricular impairment in cardiomyopathy.

## Introduction

Cardiomyopathy leads to serious congestive heart failure (CHF) with substantial risk of mortality and morbidity^1^. Cardiomyopathy is classified into dilated (DCM) and hypertrophic cardiomyopathies (HCM) according to the gross appearance of the heart. HCM can lead to CHF and is characterized by marked ventricular wall thickening, hypercontractile systolic function with diastolic dysfunction, and constitutes a major cause of sudden death in young people and athletes^2^. On the other hand, DCM is characterized by the thin myocardial wall, large ventricular chamber size, and reduced cardiac pumping ability^3^. The advancements in molecular genetics have identified a number of mutated genes responsible for both HCM and DCM^4^. Several sublines with various severities of CM arise from the most famous animal model, the Bio14.6 hamsters, outstanding among which are the TO-2 hamsters^5^. Bio14.6 hamsters exhibit marked cardiac hypertrophy and TO-2 hamsters exhibit cardiac dilation and dysfunction. It has been shown that both hypertrophic and dilated cardiomyopathies are caused by mutation of the same gene, delta-sarcoglycan, in these hamsters^6^. These findings indicate that the same genetic modifier might be involved in compensatory cardiac hypertrophy or cardiac dysfunction^7^.

Reactive oxygen species (ROS), such as superoxide anions and hydrogen peroxide, exert cytotoxic effects in the presence of oxidative stress and play an important role in the development of cardiovascular diseases, including hypertension, atherosclerosis, cardiac hypertrophy, and ischemia-reperfusion injury^8^. Moreover, increased production of ROS has been suggested to contribute to CHF^9, 10^ because oxidative stress appears to be involved in cardiac remodeling and altered calcium sensitivity^11, 12^. Indeed, the formation of ROS can be directly detected in experimental models of CHF^13, 14^ and oxidative/nitrosylative changes of proteins were largely increased in human falling hearts^15^. Among the numerous oxidative products, carbonylated proteins are the major and most commonly used oxidative modified proteins to infer oxidative stress^16, 17^. The amount of protein carbonyl groups is a good index of the extent of protein oxidative damage associated with various conditions of oxidative stress, toxicity, and diseases^18, 19^.

Identification of oxidatively damaged proteins that contribute to the pathogenesis of cardiomyopathy could help in the design of new methods for early diagnosis and finding new therapeutic alternatives for cardiomyopathy. Proteomics offers a unique mean for analysis of oxidative stress pathways at cellular level^20, 21^. Current proteomic technology allows us to examine global changes in oxidatively damaged protein levels in the diseased heart and can provide new insights into cellular mechanisms involved in cardiac dysfunction^22, 23^. Among proteomic technologies, two-dimensional gel electrophoresis (2-DE) with oxyblot analysis has sufficient resolution to separate the modification states of a protein directly including carbonylation^24^.

Understanding the cellular function underlying cardiac dysfunction is crucial to the design of novel therapies for cardiomyopathy. The aim of the present study was to measure the myocardial levels of carbonylated proteins in cardiomyopathy and to characterize ROS-induced carbonyl modification of myocardial proteins involved in cardiac dysfunction using an animal model of cardiomyopathy.

## Results

### Body weight, left ventricular (LV) function and plasma troponin T concentration

Body weight of 6-week-old hypertrophic cardiomyopathy of Syrian hamsters (Bio14.6 strain) was significantly lower than control hamsters (F1B strain) (Table 1). There was no significant difference in body weight between dilated cardiomyopathy of Syrian hamsters (TO-2 strain) and F1B hamsters. LV weight was significantly lower in Bio 14.6 than F1B control hamsters. However, there were no significant differences in LV weight/body weight among the three strains (Table 1). Because the body was small, the heart weight would be low in 6-week-old Bio 14.6 compared with other strains. To characterize LV function, we compared the echocardiographic findings in each hamster. The intra-ventricular septum was significantly lower in TO-2 and was significantly higher in Bio 14.6 than F1B control hamsters (Table 1). LV end-diastolic diameter and LV end-systolic diameter were significantly higher in TO-2 than F1B control hamsters. LV fractional shortening was also significantly lower in TO-2 than F1B control hamsters (Table 1). There were no significant differences in LV end-diastolic diameter, LV end-systolic diameter, and LV fractional shortening between Bio 14.6 and F1B hamsters. Plasma troponin T concentration was higher in TO-2 and Bio 14.6 than F1B control hamsters, but there was significant difference in only TO-2 hamsters statistically (Table 1).

**Table 1:** Body and heart weights, echocardiographic data and plasma troponin T level in 6-week-old hamsters.

	F1B	TO-2	Bio 14.6
Body weight (g)	93.0 ± 2.9	84.8 ± 2.8	81.4 ± 3.2*
LV weight (mg)	219 ± 6	198 ± 9	191 ± 7*
LV weight/body weight	2.36 ± 0.04	2.33 ± 0.05	2.35 ± 0.04
Intra-ventricular septum (mm)	1.00 ± 0.02	0.90 ± 0.01*	1.19 ± 0.01*
LV end diastolic dimension (mm)	3.89 ± 0.11	4.57 ± 0.08*	4.10 ± 0.07
LV end systolic dimension (mm)	2.34 ± 0.13	2.96 ± 0.17*	2.18 ± 0.05
Fraction shortening (%)	42.3 ± 2.1	33.0 ± 0.5*	46.1 ± 0.7
Troponin T (mg/dl)	0.45 ± 0.03	0.66 ± 0.05*	0.59 ± 0.06

Data are mean ± SEM of six hamsters per group. *P < 0.05, compared with the F1B hamsters. LV: left ventricular.

### Total protein carbonyl content and TBARS (thiobarbituric acid reactive substances) concentration

**Figure 1:**
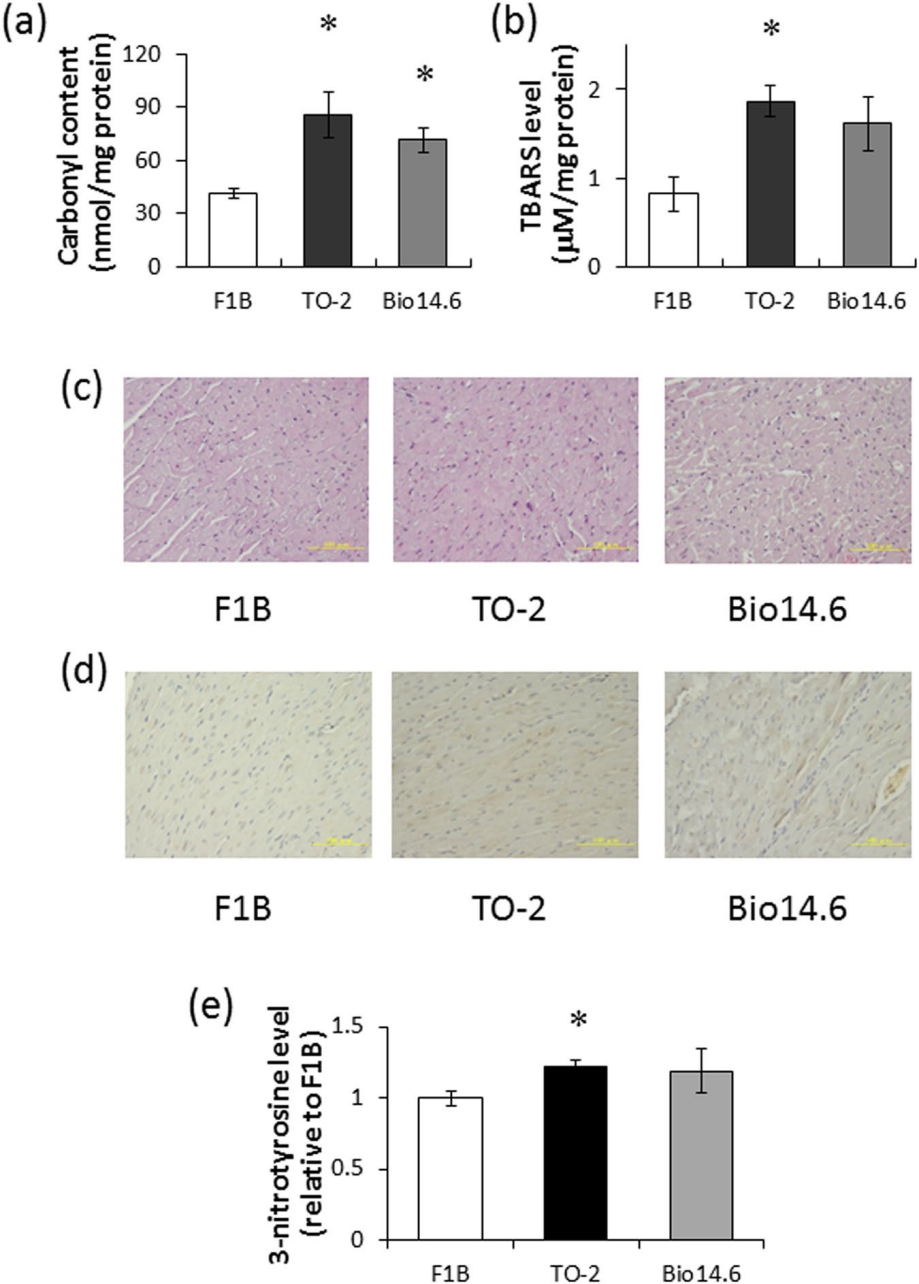
Carbonyl content, TBARS level, and morphological analysis of the left ventricles of 6-week-old hamsters. (**a**) Total protein carbonyl content and (**b**) TBARS concentration in F1B, TO-2, and Bio 14.6 hamsters. Data are mean ± SEM of six animals per group. *P < 0.05 compared with F1B hamsters. (**c**) Light micrographs of myocytes in hematoxylin-eosin stained sections of the LV wall and (**d**) Immunohistochemical staining for 3-nitrotyrosine in representative F1B, TO-2, and Bio 14.6 hamsters. Scale bars, 100 µm. (**e**) 3-Nitrotyrosine level in F1B, TO-2, and Bio 14.6 hamsters. Data are mean ± SEM of six animals per group. *P < 0.05 compared with F1B hamsters.

LV protein carbonyl content was measured to evaluate ROS-associated damage at the protein level in 6-week-old F1B, TO-2, and Bio14.6 hamsters. Total carbonyl content was significantly greater in TO-2 and Bio14.6 than F1B control hamsters (Fig. 1a). TBARS concentration was measured to reflect the level of oxidative stress. The level of TBARS was also higher in TO-2 and Bio 14.6 than F1B control hamsters, but there was significant difference in only TO-2 hamsters statistically (Fig. 1b).

### Fibrosis and 3-nitrotyrosine abundance in the left ventricle

No significant changes in the extent of interstitial fibrosis in the left ventricles were observed among the TO-2, Bio14.6, and F1B control hamsters (Fig. 1c). Immunohistochemical staining of LV sections of 6-week-old hamsters showed a marked increase in the abundance of 3-nitrotyrosine in the TO-2 and Bio14.6 hamsters compared with the F1B control hamsters (Fig. 1d), but there was significant difference in only TO-2 hamsters statistically (Fig. 1e).

### Analysis of individual proteins with increased carbonyl modification

**Figure 2:**
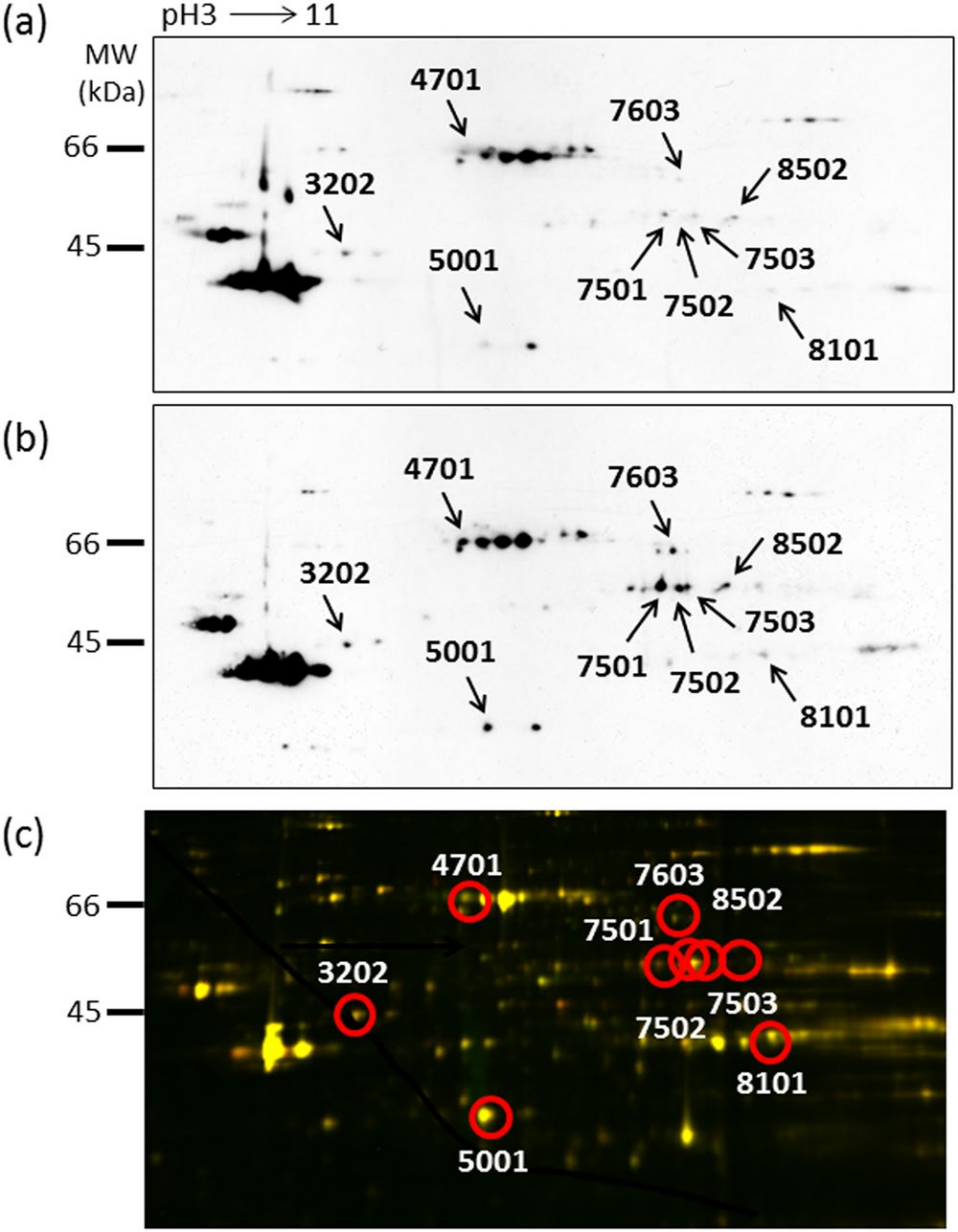
Proteomics analysis of the left ventricles of 6-week-old hamsters. A representative 2D oxyblot analysis of heart lysates in (**a**) F1B and (**b**) TO-2 hamsters. (**c**) 2D-DIGE image of heart lysates of F1B and TO-2 hamsters. The proteins (40 μg each) were labeled with Cy3 and Cy5 dyes, mixed and subjected to 2D-DIGE analysis. Cy3- and Cy5- images are illustrated using red and green pseudocolors, respectively. Yellow spots represent unchanged proteins.

**Figure 3:**
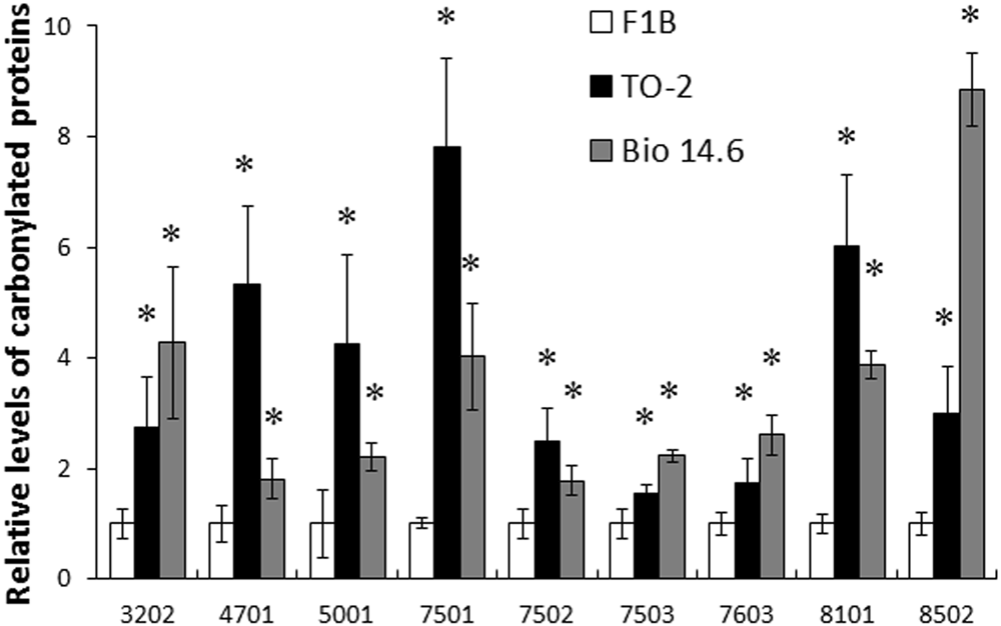
Relative levels of carbonylated proteins of the 9 spots in left ventricles of 6-week-old hamsters. Data are mean relative carbonyl protein levels ± SEM of four animals per group. *P < 0.05 compared with F1B hamsters.

2D-oxyblot analysis was performed to obtain the information on individual proteins prone to oxidation in 6-week-old F1B, TO-2, and Bio14.6 hamsters. Figure 2a and b show carbonyl proteins in representative 2D-oxyblots from F1B and TO-2 hamsters. To investigate differences in protein expression in the heart tissues, LV tissue proteins from all hamsters were also extracted and subjected to comparative analysis by two-dimensional fluorescence differential gel electrophoresis (2D-DIGE) (Fig. 2c). Eighteen spots were judged to exhibit increased carbonyl levels in TO-2 and Bio14.6 hamsters (fold change ≥1.5) compared with F1B hamsters. Finally, 9 spots were identified by MALDI-TOF-TOF/MS as follows: spot 3202 was identified as cytochrome b-c1 complex subunit 1, mitochondrial (UQCRC1); spot 4701 was identified as dihydrolipoyllysine-residue acetyltransferase component of pyruvate dehydrogenase complex, mitochondrial (DLAT); spot 5001 was identified as L-lactate dehydrogenase B chain (LDHB); spots 7501 and 7503 were identified as dihydrolipoyl dehydrogenase, mitochondrial (DLD); spot 7502 was identified as succinyl-CoA:3-ketoacid-coenzyme A transferase 1, mitochondrial (SCOT); spot 7603 was identified as electron transfer flavoprotein-ubiquinone oxidoreductase, mitochondrial (ETFDH); spot 8101 was identified as citrate synthase, mitochondrial (CS); and spot 8502 was identified as ATP synthase subunit alpha, mitochondrial (ATP1) (Table 2). Figure 3 shows the relative quantities of the 9 spots with increased carbonyl modification.

**Table 2:** List of proteins with increased carbonyl modification in hearts of TO-2 and Bio 14.5 hamsters compared with control (F1B).

Spot	Protein name	% Cov.	Peptides (95%)	Subcellular location	Biological process
3202	Cytochrome b-c1 complex subunit 1 (UQCRC1)	19.8	4	mitochondria	respiratory electron transport chain
4701	Dihydrolipoyllysine-residue acetyltransferase component of pyruvate dehydrogenase complex (DLAT)	20.0	6	mitochondria	carbohydrate metabolic process
5001	L-lactate dehydrogenase B chain (LDHB)	25.8	7	cytoplasm	carbohydrate metabolic process
7501	Dihydrolipoyl dehydrogenase (DLD)	14.6	5	mitochondria	respiratory electron transport chain
7502	Succinyl-CoA:3-ketoacid-coenzyme A transferase 1 (SCOT)	22.3	5	mitochondria	fatty acid metabolic process
7503	Dihydrolipoyl dehydrogenase (DLD)	18.6	4	mitochondria	respiratory electron transport chain
7603	Electron transfer flavoprotein-ubiquinone oxidoreductase, mitochondrial Citrate synthase (ETFDH)	11.4	4	mitochondria	respiratory electron transport chain
8101	Citrate synthase (CS)	13.4	7	mitochondria	carbohydrate metabolic process
8502	ATP synthase subunit alpha (ATP1)	22.1	8	mitochondria	ATP biosynthetic process

### Functional categories and expressional location of identified proteins

To understand their biological roles, the 9 proteins with increased carbonyl modification were assigned to protein analysis through an evolutionary relationships (PANTHER) database. Eight of the 9 proteins were located in the mitochondria (Table 2). The PANTHER classification system indicated that the 9 proteins with increased carbonyl modification could be classified into four groups according to their functional properties: *1)* respiratory electron transport chain, 2*)* carbohydrate metabolic process, *3)* fatty acid metabolic process, and *4)* ATP biosynthetic process (Table 2).

### SCOT, citrate synthase, and ATP assay

**Figure 4:**
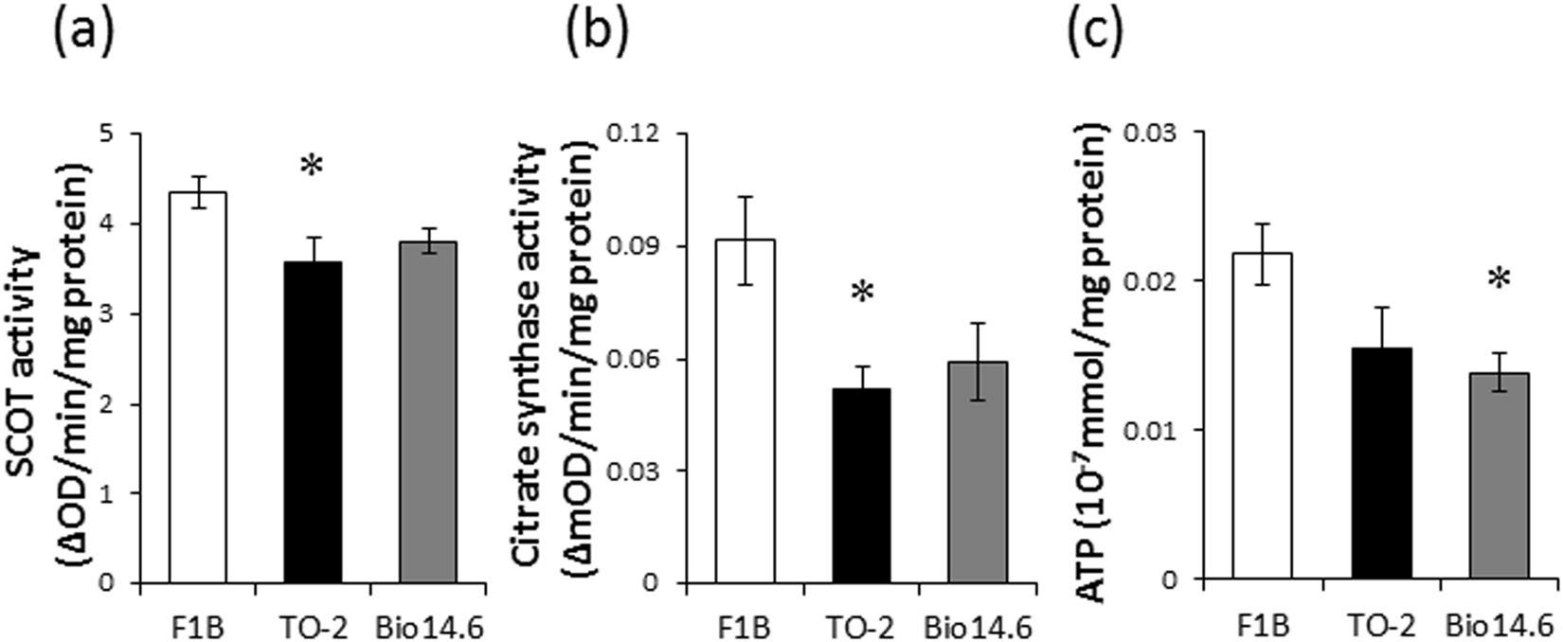
SCOT and citrate synthase activities and ATP content. (**a**) SCOT and (**b**) citrate synthase activities as well as (**c**) ATP content of left ventricles of F1B, TO-2, and Bio 14.6 hamsters. Data are mean ± SEM of six animals per group. *P < 0.05 compared with F1B hamsters.

**Figure 5:**
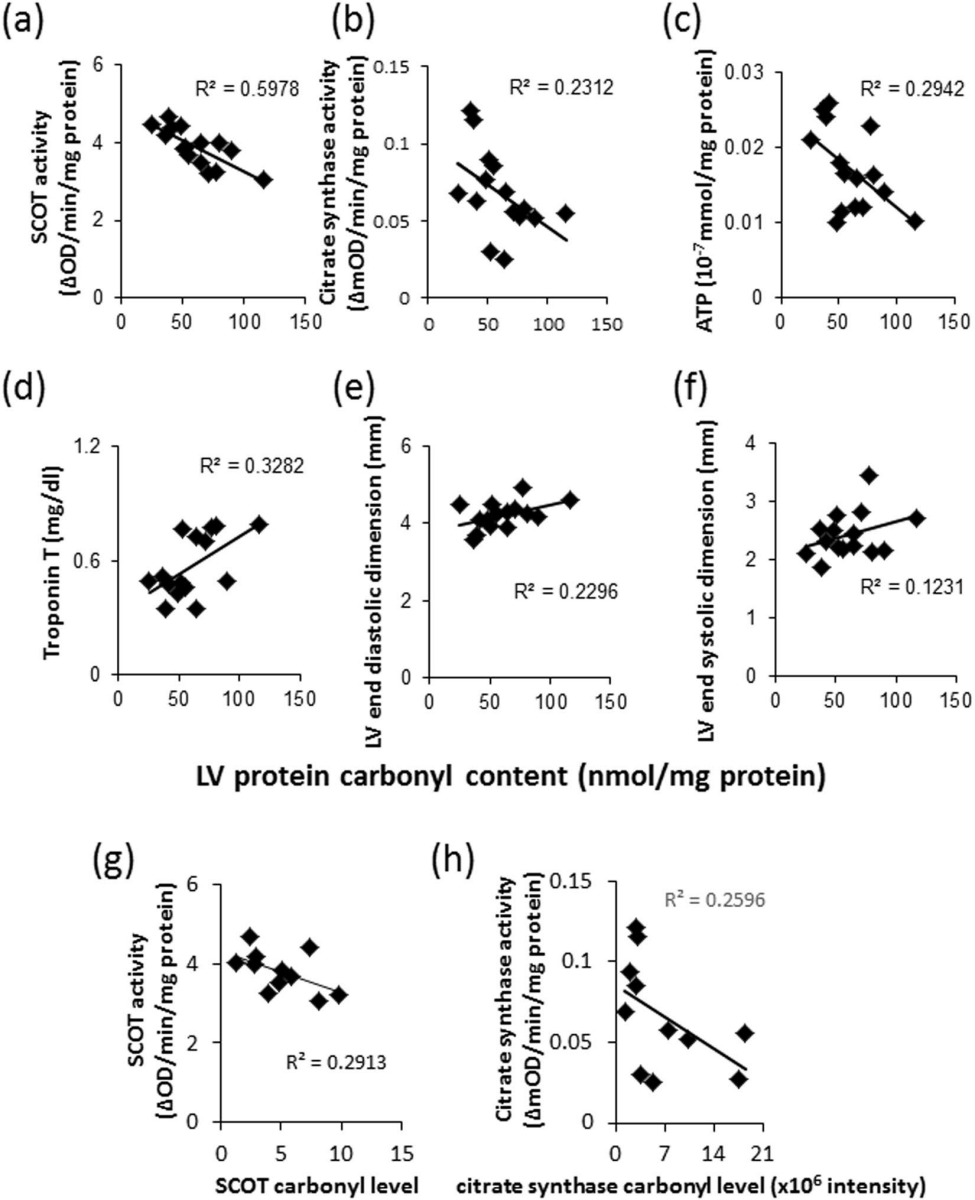
Correlation of carbonyl contents with the biomolecules and cardiac function. Correlation between LV protein carbonyl contents and (**a**) SCOT activity, (**b**) citrate synthase activity, (**c**) ATP content, (**d**) plasma Troponin T level, (**e**) LV end-diastolic diameter, and (**f**) LV end-systolic diameter. Data are mean ± SEM of six animals per group. Correlation between relative carbonyl levels calculated from the 2D-oxyblot and 2D-DIGE analyses and activities of (**g**) SCOT and (**h**) citrate synthase. Data are mean ± SEM of four animals per group.

To evaluate whether protein carbonylation is associated with alterations in enzymatic activity, we measured the SCOT and CS activities as well as ATP concentration in the left ventricles of hamsters of the three groups. The SCOT and CS activities were significantly lower in the left ventricles of TO-2 hamsters than the control hamsters (Fig. 4a,b). The level of ATP was significantly lower in the left ventricles of Bio 14.6 hamsters than the control hamsters (Fig. 4c). Further analysis indicated that LV carbonyl production correlated significantly and negatively with SCOT activity (*r* ^2^ = 0.60, *P* = 0.0007; Fig. 5a) and ATP concentration (*r* ^2^ = 0.30, *P* = 0.037; Fig. 5c) whereas there were no significant correlation with CS activity (*r* ^2^ = 0.23, *P* = 0.070; Fig. 5b). We also analyzed the correlation between carbonyl production and plasma Troponin T level, as well as functional parameters. As the results, we observed a significant correlation between carbonyl production and Troponin T level (*r* ^2^ = 0.33, *P* = 0.026; Fig. 5d). However, there were no significant correlation between carbonyl production and cardiac functions, such as LV end-diastolic diameter (*r* ^2^ = 0.23, *P* = 0.070; Fig. 5e), LV end-systolic diameter (*r* ^2^ = 0.12, *P* = 0.29; Fig. 5f), or LV fractional shortening (*r* ^2^ = 0.029, *P* = 0.85). We also analyzed the correlation between relative carbonyl levels calculated from the 2D-oxyblot and 2D-DIGE analyses and activities of SCOT and CS. The relative carbonyl level of SCOT or CS tended to correlate with the activity of SCOT (*r* ^2^ = 0.29, *P* = 0.087; Fig. 5g) or CS (*r* ^2^ = 0.26, *P* = 0.11; Fig. 5h). However, there were no significant correlations statistically.

## Discussion

We performed 2D-oxyblot analysis and identified 9 proteins with increased carbonyl level in cardiomyopathy hamsters compared with control hamsters. Almost all carbonylated proteins identified in cardiomyopathy hamsters were mitochondrial proteins and related to ATP synthesis. In addition, protein carbonylation had negative effects on SCOT activity and ATP concentration. Decrease in ATP concentration could be a consequence of protein/enzyme inactivation related to ATP synthesis. Mitochondria are the major source for energy supply in cardiomyocytes and cardiac performance depends on the energy produced by these organelles^25^. Murray *et al*.^26^ described profound abnormalities in myocardial energy metabolism in the failing heart, and concluded that these abnormalities correlate with clinical symptoms and survival. Since myocardial mitochondrial abnormities lead to metabolic remodeling, deficit in cardiac energetic, with eventual dysfunction of the myocardium^27^, the reduction in energy metabolism by oxidative damage could be involved in LV impairment in cardiomyopathy. Balogh *et al*.^28^ have observed significantly higher carbonylation levels of myofilament proteins, such as myosin heavy chain and actin in the infarcted LV region of mouse hearts. The present study did not observe the changes in the levels of carbonylation of any myofilament proteins because all the contractile proteins that were less soluble might not be extracted.

Oxidative stress appears to be involved in cardiac remodeling and altered calcium sensitivity, and ROS seem to play an important role in the development of cardiovascular diseases^10^. Protein carbonyl formation is a major marker of protein oxidation, which can arise from direct free radical attack on amino acid side chains^29^. In the present study, protein oxidation was assessed by measuring protein carbonyl content. The results showed significantly high total protein carbonyl content in 6-week-old cardiomyopathy hamsters compared with age-matched control hamsters, indicating early induction of oxidative stress and protein damage in left ventricles of both DCM and HCM hamsters. We also measured 3-nitrotyrosine level and TBARS concentration in the LV homogenates and compared the values among three groups. 3-Nitrotyrosine is a product of tyrosine nitration mediated by reactive nitrogen species such as peroxynitrite anion and nitrogen dioxide, and TBARS is the product to evaluate lipid peroxidation. The levels of 3-nitrotyrosine and TBARS were higher in TO-2 and Bio 14.6 than F1B control hamsters, but there were significant differences in only TO-2 hamsters statistically. However, the tendency of increased values of two parameters was consistent with the cardiac carbonylation production among three groups, suggesting that carbonylation is oxidative protein damage that is one of the leading causes of myocardial injury^30^.

**Figure 6:**
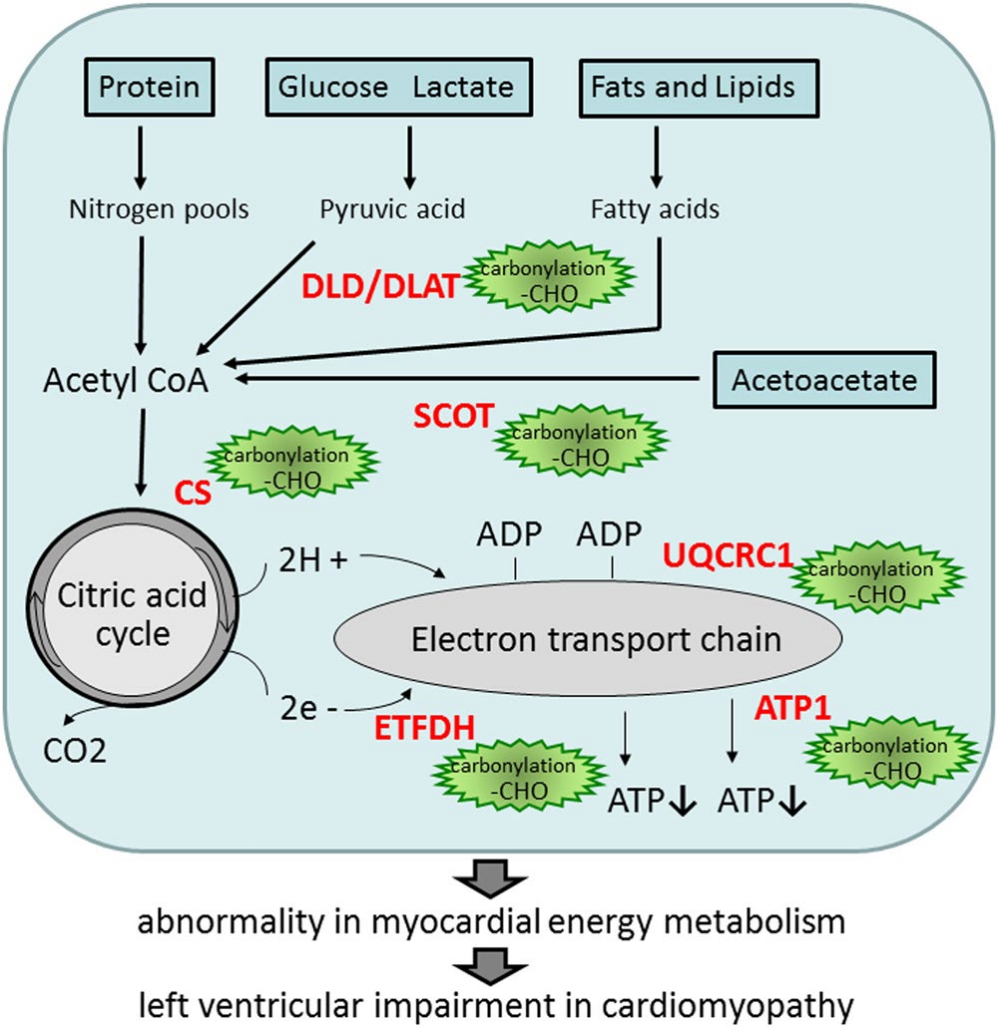
Schematic representation of the pathways. The pathways of myocardial substrate metabolism, including the molecules with increased carbonyl levels in TO-2 and Bio 14.6 hamsters compared with F1B hamsters.

To assess the cardiac protein oxidation in cardiomyopathy, we used 2D-oxyblot analysis to identify specific targets of protein oxidation in left ventricles. UQCRC1, DLAT, DLD, SCOT, ETFDH, CS, and ATP1 were located in the mitochondria. Figure 6 is a schematic representation of the pathways of myocardial substrate metabolism, including the above molecules. DLAT and DLD are enzyme components of the multienzyme pyruvate dehydrogenase complex (PDC). PDC regulates the entry of glycolytic products into the tricarboxylic acid cycle by catalyzing oxidative decarboxylation of pyruvate to acetyl-CoA in the mitochondria of mammalian cells^31^. SCOT is a rate-determining enzyme in the degradation of ketone bodies, which occurs in the mitochondria of many extrahepatic organs and in the process of ketone body acetoacetate conversion to acetyl-CoA^32^. CS is a pace-making enzyme in the first step of the citric acid cycle (or Krebs Cycle)^33^. CS is localized within eukaryotic cells in the mitochondrial matrix and catalyzes the condensation reaction of the two-carbon acetate residue from acetyl-Co A and a molecule of four-carbon oxaloacetate to form the six-carbon citrate^34^. Finally, UQCRC1 is a component of the ubiquinol-cytochrome c reductase complex, which is part of the mitochondrial respiratory chain. ETFDH is essential for electron transfer from a number of mitochondrial flavin-containing dehydrogenases to the main respiratory chain. ATP1 is a subunit of mitochondrial ATP synthase, which is an enzyme that creates the energy storage molecule ATP^35^. Oxidative changes in these proteins impair energy production, suggesting that mitochondrial dysfunction is involved in the development of cardiac dysfunction in cardiomyopathy (Fig. 6).

The present study showed a negative correlation between LV carbonyl production and SCOT activity. In this regard, SCOT activity is known to be decreased in the heart mitochondria of diabetic rats^36^. Furthermore, SCOT is the main prominent target of nitration^37^ and SCOT nitration was also reported to be relatively higher and SCOT activity to be lower in inflammation and hyperglycemia^38^. In our study, carbonyl level of SCOT was increased and SCOT activity was decreased in the hearts of cardiomyopathy hamsters. We analyzed the correlation between the activity of SCOT and the relative carbonyl level of SCOT calculated from the 2D-oxyblot and 2D-DIGE analyses, but there were no significant correlations statistically (*r* ^*2*^ = 0.29, *P* = 0.087). We need further studies to confirm whether the carbonylation of SCOT regulates SCOT activity. The cardiac muscle produces energy from a wide range of substrates; including fatty acids, glucose, lactate, and ketone bodies^39^. Our findings suggest a shift in the source of acetyl-CoA to the citric acid cycle in cardiomyopathic hearts. In addition, we also found a negative correlation between LV carbonyl production and ATP concentration. Oxidative modification of proteins involved in the citric acid cycle and electron transport chain might contribute to the abnormality in myocardial energy metabolism and lead to LV impairment in cardiomyopathy.

Oxidative protein damage has been implicated as one of the leading causes of myocardial injury. To identify the ROS-induced carbonyl modification of proteins in left ventricles of animals with cardiomyopathy, we used 6-week-old cardiomyopathy hamsters. After the phenotype becomes obvious as cardiac dysfunction or hypertrophy, it is difficult to know whether carbonyl modification in left ventricles of cardiomyopathy is the cause or the result of cardiac impairment. Therefore, we used younger mice whose phenotypes of cardiomyopathy had not been manifested. Sakamoto^40^ has shown that the cardiac function was impaired in 6-week-old TO-2 hamsters, but the phenotype was not established yet in the present study. The present study identified the proteins with increased carbonyl level in cardiomyopathy hamsters compared with control hamsters. The present study also showed a significant correlation between carbonyl production and Troponin T level. Troponin T is a biomarker predicting of the early stage of cardiac impairment^41^. Indeed, our previous study showed that diastolic cardiomyopathy of hamsters (TO-2 strain) manifested severe LV dysfunction after 8 weeks old, and plasma Troponin T levels were significantly increased in 8-week-old TO-2 hamsters compared with 4-, 12-, 18-, or 32-week-old TO-2 hamsters^42^. Thus, we believe that the observation of present study suggests that carbonyl modification was associated with the development of cardiac impairment. However, we need further studies to confirm whether oxidative protein damage: carbonylation directly lead to cardiac impairment by long term observation using inhibition or enhancement experiment models.

In conclusion, we applied 2D-oxyblot proteomic analysis in the present study to identify the molecular targets in left ventricles of cardiomyopathy. The results showed carbonylation of proteins related to ATP synthesis, including citric acid cycle and electron transport system in the hearts of hamsters with cardiomyopathy. These findings suggest that oxidative stress-induced reduction in energy metabolism seems to lead, at least in part, to LV impairment and contribute to the pathogenesis of cardiomyopathy.

## Methods

### Experimental animals

Male dilated cardiomyopathy (TO-2 strain) and hypertrophic cardiomyopathy (Bio14.6 strain) of Syrian hamsters and control hamsters (F1B strain) were obtained from BIO Breeders (Fitchburg, MA)^42^. Hamsters were sacrificed at 6 weeks of age. The investigation conformed to the Guide for the Care and Use of Laboratory Animals published by the US National Institutes of Health (NIH Publication No. 85-23, revised 1996) and was approved by the Committee on Laboratory Animals Utilization of Mie University. All animals were housed in an air-conditioned facility set at 22–24 °C with 12:12 h light/dark cycles and free access to food and water.

### Physiological measurements and biochemical tests

Before sacrifice, the animals underwent transthoracic echocardiography using the HD 11 XE system (Philips Healthcare, Amsterdam, Netherlands) as described previously^43^. The hamsters were anesthetized by intraperitoneal injection of 50 mg/kg body weight of pentobarbital sodium. Wall thickness and LV end-diastolic diameter were obtained from the short-axis view at the level of the papillary muscles, and LV fractional shortening was calculated. Blood was collected from anesthetized animals, transferred to a chilled tube containing heparin, and centrifuged. Plasma was stored at −80 °C until analysis. Serum level of troponin T was measured by rat Troponin T ELISA kit (TSZ ELISA, Framingham, MA).

### Extraction of proteins from the myocardium

The heart tissues were removed and immediately frozen in liquid nitrogen and stored at −80 °C. Frozen tissues were homogenized in lysis buffer (30 mM Tris-HCl, 7 M urea 2 M thiourea, 4% w/v CHAPS, and a cocktail of protease inhibitors, pH 8.5). After incubation for 60 min on ice, homogenates were centrifuged at 30,000 × g for 30 min at 4 °C and the supernatants were collected for proteomic analysis. The protein concentration in the supernatants was determined using the Pierce 660 nm Protein Assay Kit (Thermo Fisher Scientific, Waltham, MA) with bovine serum albumin as a standard.

### Determination of carbonyl content and TBARS Level

The levels of total protein carbonyls, an indicator of protein oxidation, were measured using the Protein Carbonyl Assay Kit (Cayman Chemical, Ann Arbor, MI). The assay is based on the reaction of 2, 4-dinitrophenylhydrazine (DNPH) with protein carbonyls to form hydrazones, which can be quantified spectrophotometrically, using the Benchmark Plus multiplex plate reader (Bio-Rad Laboratories, Hercules, CA). The amount of TBARS in the homogenates of heart was measured by the TBARS assay kit (Cayman Chemical). Absorbance was determined at a wavelength of 370 nm or 530 nm, respectively, using the plate reader.

### Histology and immunohistochemistry

Midventricular slices were fixed in 4% paraformaldehyde, embedded in paraffin, sectioned at a thickness of 5 μm, and stained with hematoxylin eosin solution. Sections were also incubated for 20 min at room temperature with 10% normal goat serum in phosphate-buffered saline and then subjected to immunohistochemistry with rabbit polyclonal antibody to 3-nitrotyrosine (1:100 dilution; Upstate Biotechnology, Lake Placid, NY), as described previously^13^. The area of 3-nitrotyrosine staining was calculated using CellSens Imaging Software (Olympus, Tokyo, Japan) and was then compared with the mean area on sections of F1B (defined as 1.0).

### Two-dimensional fluorescence differential gel electrophoresis (2D-DIGE)

Heart proteins (*n* = 6, each group) were labeled with amine-reactive cyanine dyes, Cy3 or Cy5 developed for fluorescence 2D-DIGE technology (GE Healthcare, Little Chalfont, UK)^44^. Internal pools were generated by combining equal amounts of all samples and were labeled with Cy2. Then, 2-DE was performed, as described previously^45^. After 2-DE, cyanin-labeled proteins were visualized directly by scanning, using a Typhoon 9400 imager (GE Healthcare) in fluorescence mode. Using DeCyder Differential Analysis Software (DeCyder software V6.0, GE Healthcare), abundance measurements for each gel by comparing normalized volume ratios of individual spots from Cy3- or Cy5-labeled samples to the corresponding Cy2-signals from the pooled samples (internal standard) were carried out. Thereafter, all gel comparisons and initial screening type statistical analyses were performed with the biological variation analysis module.

### Detection of carbonyl modification of proteins (2D-oxyblot analysis)

To detect individual carbonyl-modified proteins, heart proteins (*n* = 4, each group) were precipitated and cleaned using a 2-D Clean-up Kit (GE Healthcare), according to the instructions provided by the manufacturer. Carbonylated proteins were labeled by derivatization of carbonyl group with 2,4-denitrophenylhydrazone (DNP) by reaction with DNPH. To reduce artefactual oxidation of the samples, we added thiourea in lysis buffer. Because we homogenized from tissue in the same way and derivatized all the samples on the same day, we believe that there were no differences in the degree of artefactual oxidation, even if it occurred, among three groups. Then, 2-DE and immunoblotting using an antibody specific to the DNP moiety (Oxyblot Protein Oxidation Detection Kit, Millipore, Billerica MA) were performed as described previously^46^. In brief, the derivatized samples were applied to Immobiline DryStrips (24 cm, non-linear pH 3–11; GE Healthcare) for the first-dimension isoelectric focusing. After reduction and alkylation of disulfide bonds with 10 mg/ml DTT and 25 mg/ml iodoacetamide, respectively, the second-dimension 12.5% SDS–polyacrylamide gel electrophoresis was run on an Ettan DALT Six large electrophoresis system (GE Healthcare). After the second-dimension, the proteins from the gels were transferred to polyvinylidene fluoride (PVDF) membrane (Immobilon-P transfer membrane; Millipore) using a TE77 semidry transfer unit (GE Healthcare) at 50 V for 30 min. The 2,4-DNP adduct of the carbonyls of the proteins was detected on the PVDF membrane using a primary rabbit antibody specific for DNP–protein adducts (1:250; Millipore), and a secondary horseradish peroxidase conjugated goat anti-rabbit IgG antibody (1:500; Millipore) was applied. Then, the spot intensities of carbonyl proteins were quantified using PDQuest version 8.0 Advanced 2-D Analysis software (Bio-Rad Laboratories). We calculated specific oxidation as the relative values of carbonyl level (obtained from the 2D-oxyblot analysis) per protein expression (obtained from the 2D-DIGE analysis).

### Protein identification

Protein spots with *p* ≤ 0.05 (two-tailed Student’s *t*-test) and a fold change ≥1.5 in all three comparisons were chosen for further identification. 2-DE was performed according to the 2D-DIGE described procedure and using 12.5% acrylamide slab gels. Following 2-DE, gels were stained with Coomassie Brilliant Blue (CBB) R-350 (PhastGel Blue R, GE Healthcare) and matched to the immunoblotting images. Selected spots were excised manually from CBB-stained preparative -2DE gels with gel spot cutter. Then, in-gel digestion of protein samples was performed using the protocol described previously^45^. Mass analysis of peptide mixtures was performed using a matrix-assisted laser desorption ionization time-of-flight tandem mass spectrometry (MALDI-TOF/TOF MS; 4800 *Plus* MALDI TOF/TOF^TM^ Analyzer; Life Technologies, Carlsbad, CA) operating in positive-ion reflector mode. The obtained peptide mixture was deposited onto the MALDI target closely followed by matrix solution containing 50% α-cyano-4-hydroxycinnamic acid (Wako Pure Chemical Industries, Osaka, Japan). A protein database search was performed with Paragon Method using Protein Pilot software (Life Technologies) to identify excised proteins.

### Measurement of levels of citrate synthase, SCOT, and ATP

Protein samples were prepared according to instructions provided by the manufacturer and the citrate synthase activity in heart tissues was assayed by the Citrate Synthase Activity Assay Kit (Abcam, Cambridge, MA). In brief, the samples (100 μl) were added to the pre-coated microplate strips, sealed, and incubated for 3 hours at room temperature. The wells were aspirated and washed twice with wash buffer. Activity solution (100 μl) was then added to each well and the plate was read every 5 min for 60 min at a wavelength of 412 nm using the plate reader. The activity of succinyl-CoA: 3-ketoacid coenzyme A -transferase was measured as described previously^47^ with modification. The assay mixture consisted of 100 mM Tris/HCl (PH 8.5), 10 mM MgCl_2_, 4 mM iodoacetamide, 0.2 mM succinyl-CoA, and 50 mM acetoacetate. Samples (5–25 μg/10 μl) were added to the 1 ml of the assay mixture and the change in absorbance was recorded every 1 min for 5 min at a wavelength of 313 nm. The amount of ATP in the homogenates of heart was measured by the ATP assay kit (Wako Pure Chemical Industries). In brief, LV tissues were homogenized with cold lysis buffer (0.25 M sucrose and 10 mM HEPES, PH 7.4) and then centrifuged at 1,000 × g at 4 °C for 10 min. The samples (1 ml) were added to 7 ml of lysis buffer. The diluted samples (100 μl) were mixed with the same volume of ATP reagent and incubated for 30 min at room temperature. L/L reagent (100 μl) was then added to the mixtures (100 μl) and data were determined by luminescence (GLOMAX 20/20 luminometer; Promega, Madison, WI). All levels were normalized to protein concentration.

### Statistical analysis

Data are expressed as mean ± standard error of the mean (SEM). Comparisons among three groups were tested using one-way analysis of variance (ANOVA) followed by Dunnett’s multiple comparison tests. Pearson’s correlation coefficient analysis was performed with the levels of citrate synthase, SCOT, and ATP for heart protein carbonyl. All statistical analyses were performed using the JMP 8.0 software (SAS Institute Inc, Cary, NC). A *P* value < 0.05 was considered statistically significant.
